# Quantitative Evaluation of Enhancement Patterns in Focal Solid Liver Lesions with Gd-EOB-DTPA-Enhanced MRI

**DOI:** 10.1371/journal.pone.0100315

**Published:** 2014-06-20

**Authors:** Michael Haimerl, Max Wächtler, Florian Zeman, Niklas Verloh, Ivan Platzek, Andreas Georg Schreyer, Christian Stroszczynski, Philipp Wiggermann

**Affiliations:** 1 Department of Radiology, University Medical Center Regensburg, Regensburg, Germany; 2 Center for Clinical Trials, University Medical Center Regensburg, Regensburg, Germany; 3 Department of Radiology, University Hospital Carl Gustav Carus Dresden, Dresden, Germany; Northwestern University Feinberg School of Medicine, United States of America

## Abstract

**Purpose:**

The objective was to investigate the dynamic enhancement patterns in focal solid liver lesions after the administration of gadolinium ethoxybenzyl diethylenetriamine pentaacetic acid (Gd-EOB-DTPA) by means of dynamic magnetic resonance imaging (MRI) including hepatobiliary phase (HP) images 20 min after Gd-EOB-DTPA administration.

**Materials and Methods:**

Non-enhanced T1/T2-weighted as well as dynamic magnetic resonance (MR) images during the arterial phase (AP), the portal venous phase (PVP), the late phase (LP), and the HP (20 min) were obtained from 83 patients (54 male, 29 female, mean age 62.01 years) with focal solid liver lesions. MRI was conducted by means of a 1.5-T system for 63 patients with malignant liver lesions (HCCs: n = 34, metastases: n = 29) and for 20 patients with benign liver lesions (FNH lesions: n = 14, hemangiomas: n = 3, adenomas: n = 3). For quantitative analysis, signal-to-noise ratios (SNR), contrast enhancement ratios (CER), lesion-to-liver contrast ratios (LLC), and signal intensity (SI) ratios were measured.

**Results:**

The SNR of liver parenchyma significantly increased in each dynamic phase after Gd-EOB-DTPA administration compared to the SNR of non-enhanced images (p<0.001). The CER of HCCs and metastases significantly decreased between LP and HP images (p = 0.0011, p<0.0001). However, FNH lesions did not show any significant difference, whereas an increased CER was found in hemangiomas. The mean LLCs of FNH lesions were significantly higher than those of HCCs and metastases. The LLC values of hemangiomas remained negative during the entire time course, whereas the LLC of adenomas indicated hyperintensity from the AP to the LP. Furthermore, adenomas showed hypointensity in HP images.

**Conclusion:**

Gd-EOB-DTPA-enhanced MRI may help diagnose focal solid liver lesions by evaluating their enhancement patterns.

## Introduction

The detection and correct differentiation of focal solid liver lesions still represents a challenge in daily clinical routine. However, accurate diagnosis of such lesions is crucial for choosing therapeutic approaches, tumor therapies, and surgical interventions. Several trials have shown the superiority of magnetic resonance imaging (MRI) to ultrasonography (US) and computed tomography (CT) in both the detection and diagnosis of focal solid liver lesions, and this superiority is mainly caused by the superior soft tissue contrast of MRI [1], [2]. Therefore, dynamic contrast-enhanced MRI has been the method of choice for diagnosing focal solid liver lesions [3]–[5]. Over the past few years, much effort has been put into technical advances, such as improving software and hardware, and into the development of new magnetic resonance (MR) liver-specific contrast agents for improving the diagnostic performance of MR liver images. The contrast agent Gadolinium ethoxybenzyl diethylenetriaminepentaacetic acid (Gd-EOB-DTPA, gadoxetic acid disodium, Primovist, Schering, Berlin, Germany) has only been recently introduced into clinical practice for hepatic MRI examinations [6], [7]. Gd-EOB-DTPA has extracellular properties similar to that of conventional Gadolinum-containing extracellular MRI contrast agents. However, Gd-EOB-DTPA has an additional property, i.e. an active ATP-dependent hepatocyte uptake in which approximately 50% of the injected dose is taken up via the organic anion transporter protein 1 (OATP1) and excreted by the biliary route [6], [8]. Therefore, Gd-EOB-DTPA combines the properties of initial tumor perfusion in dynamic images and enhancement of delayed images in tumors with a large blood pool or with hepatocytes maintaining cell membrane function. Gd-EOB-DTPA-enhanced hepatobiliary phase (HP) MRI has only recently been shown to facilitate the differential diagnosis of hepatocellular lesions with and without functioning bile ducts. Hepatocellular lesions with functioning bile ducts, such as focal nodular hyperplasia (FNH), exhibit iso- or hyperintensity during the hepatobiliary phase, whereas hepatocellular lesions without any bile ducts, such as adenomas (HCA) and most hepatocellular carcinomas (HCC), lack enhancement in the hepatobiliary phase [9]–[11]. However, these trials evaluated the qualitative appearance of focal solid liver lesions during Gd-EOB-DTPA-enhanced MRI. Moreover, only very limited data exist both on the quantification of enhancement patterns of solid focal liver lesions after the injection of Gd-EOB-DTPA as well as on the potential to distinguish different lesions.

The purpose of this retrospective trial was to quantitatively evaluate the enhancement patterns of solid focal liver lesions after the administration of Gd-EOB-DTPA with the aim of differentiating liver lesions according to their enhancement characteristics.

## Materials and Methods

### Patients

This retrospective trial was conducted from January 2009 to September 2010 and included 83 patients with suspicious solid focal liver lesions detected with US examinations or contrast-enhanced CT. Patients underwent hepatic Gd-EOB-DTPA-enhanced MRI to confirm or rule out malignancy. None of the patients had any history of prior thermal ablation or chemotherapy including transcatheter arterial chemoembolization (TACE). None of the patients suffered from hemosiderosis or hemochromatosis because these conditions may possibly change the signal intensity (SI) of the liver parenchyma due to iron deposition and a consecutively altered liver-to-lesion contrast ratio (LLC). Our study group comprised 54 men (mean age: 66.7; age range: 43 to 86 years) and 29 women (mean age: 53.3; age range: 21 to 83 years).

### Ethics statement

The ethics committee of University of Regensburg confirmed, that for this retrospective study without any study-related clinical intervention or use of patients' personal data no ethics-approval or commission's opinion was necessary. Patient information was anonymized and de-identified prior to analysis.

### Standard of reference

Lesions were considered benign (n = 20) if they did not show any interval change of their size in the follow-up CT or MRI examination after 6 months without treatment.

**Figure 1:**
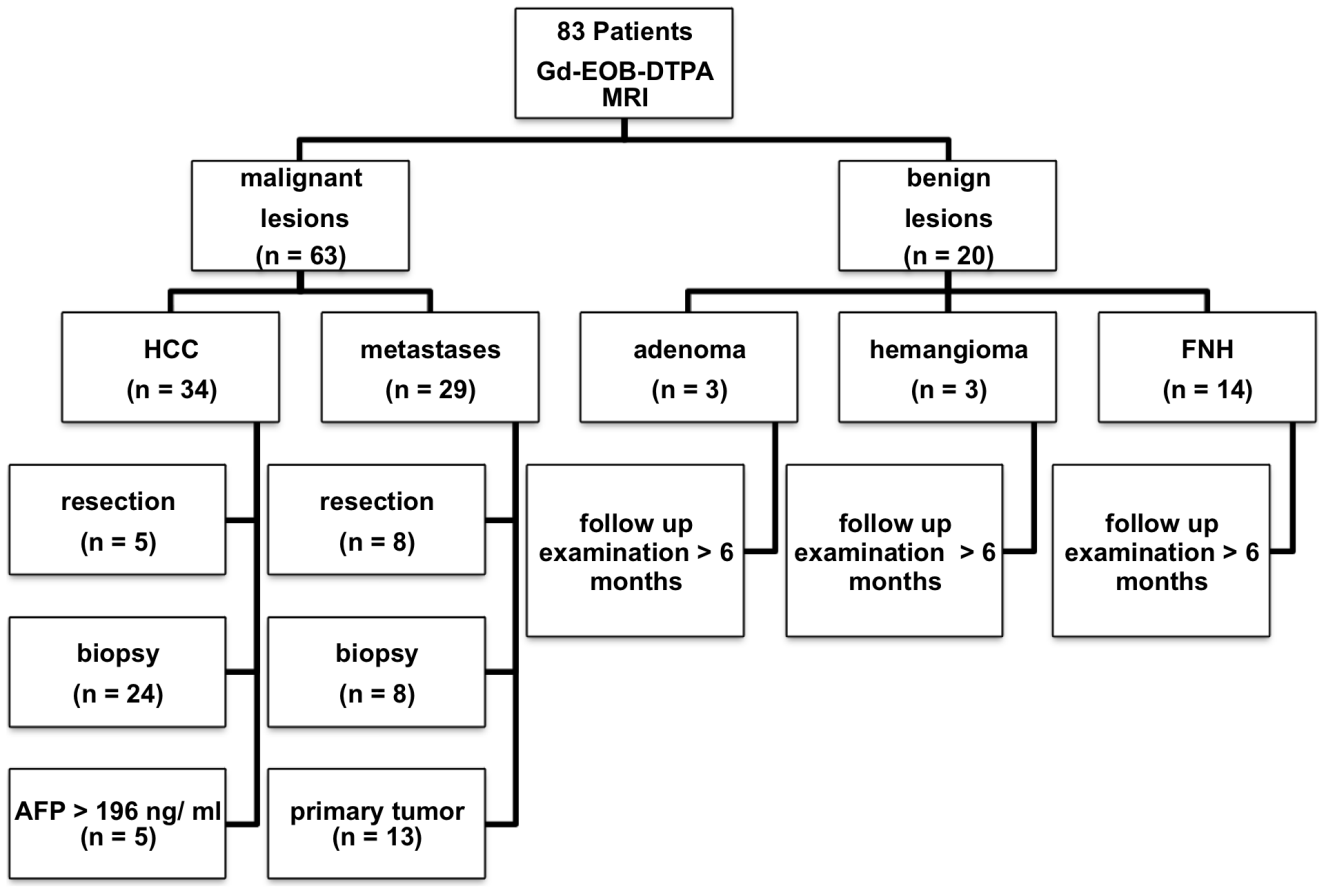
Flowchart of patients and lesions. 83 consecutive patients were included in this trial. Malignant lesions (n = 63) were either histologically proven (n = 45), or the diagnosis was based on AFP>196 ng/ml (n = 5) or on the knowledge of the primary tumor in case of metastases (n = 13). Benign lesions (n = 20) did not show any change during the follow-up examinations over more than 6 months.

Diagnosis of malignant liver lesions was confirmed by means of intraoperative findings and either consecutive histology (HCCs, n = 5; metastases, n = 8), percutaneous needle biopsy (HCCs, n = 24; metastases, n = 8), or surveillance by cross-sectional imaging either with known primary tumor (metastases, n = 13) or with pathologically elevated tumor marker (α-fetoprotein (AFP) >196 ng/ml, HCC, n = 5). 15 patients with HCC were diagnosed with liver cirrhosis (Child Pugh A, n = 11; Child Pugh B, n = 3, Child Pugh C, n = 1). Fig. 1 summarizes the means of confirming the diagnosis of a liver lesion.

### MR Imaging

Gadoxetic acid-enhanced MRI examinations were conducted by means of a 1.5 T system (Magnetom Symphony, Siemens, Erlangen, Germany) with the manufacturer's body and spine array coils. The entire liver was imaged in the transverse plane.

The pre-contrast protocol consisted of the following sequences: respiratory-triggered single-shot T2-weighted turbo spin-echo images (repetition time/echo time: 1000/85; slice thickness: 6 mm; matrix: 180×320; flip angle: 150°) followed by two different breath-hold fast-spoiled gradient-echo images, i.e. T1-weighted in-phase (repetition time/echo time: 87/4.8; slice thickness: 6 mm; matrix: 154×320; flip angle: 60°) and T1-weighted out-of-phase images (repetition time/echo time: 100/2.7; slice thickness: 6 mm; matrix: 154×320; flip angle: 70°). Then, a three-dimensional T1-weighted gradient-echo sequence was conducted using the fat suppression technique (repetition time/echo time: 4.0/1.5; slice thickness: 6 mm; matrix: 174×320; flip angle: 10°). After the administration of 10 ml Gd-EOB-DTPA with an infusion rate of 3 mL/s via a 22-gauge intravenous cubital line followed by a 15 mL saline-flush, the latter sequence was repeated 15 s, 60 s, and 120 s (dynamic phases) as well as 20 min after the contrast injection to obtain dynamic contrast-enhanced MR images. Also, respiratory-triggered T2-weighted turbo spin-echo images with fat suppression (repetition time/echo time: 2220/79; slice thickness: 6 mm; matrix: 320×320; flip angle: 140°) and respiratory-triggered diffusion-weighted images (repetition time/echo time: 1900/72; slice thickness: 6 mm; matrix: 144×192) were obtained between the late phase (LP) after 120 s and the HP as part of the routine liver MRI protocol.

### Imaging analysis

For the image analysis, we used the commercially available workstation of MRI scanner (Magnetom Symphony, Siemens, Erlangen, Germany) and did the quantitative analysis of the reference lesions with regard to the histology of tumor lesions, laboratory results, patient histories, and the findings on previous cross-sectional imaging in a blinded manner. SI of liver parenchyma, reference focal solid liver lesions, and background noise was measured in each patient for unenhanced T1-weighted gradient-echo sequence, unenhanced respiratory-triggered single-shot T2-weighted turbo spin-echo, and dynamic T1-weighted gradient-echo sequences after 15 s during the arterial phase (AP), after 60 s during the portal venous phase (PVP), after 120 s during the LP, and after 20 min during the HP. Background noise was defined as the standard deviation (SD) of the SI that was measured in the air outside the body at the position of the intrahepatic region of interest (ROI); thus, artifacts resulting from breathing and vascular pulsing could be avoided. Comparable slice positions and identical intra-axial positions were chosen. To measure SI of normal liver parenchyma, ROIs were placed in such a manner that blood vessels, necrotic areas, critical tissue, and artifacts were avoided. For each sequence, ROIs in liver parenchyma and focal solid liver lesions were drawn as large as possible (range: 0.7–7 cm).

Signal-to-noise ratio (SNR) of the liver parenchyma was calculated as SI_LV_/SI_A_. SI_LV_ is the SI of the liver and SI_A_ is the standard deviation of the SI of the air used as a background noise. LLC was calculated as follows: (SI_LE_ – SI_LVE_)/SI_A_. SI_LE_ is SI of the lesion on enhanced images, and SI_LVE_ is the SI of the liver on enhanced images. Contrast enhancement ratios (CER) in focal solid liver lesions were calculated as follows: (SI_LE_ - SI_LU_)/SI_LU_ x 100, in which SI_LU_ represents the SI of the lesion on unenhanced images. The SI ratio of each tumor in dynamic phase images was also calculated as SI_LE_/SI_LVE._ All images were plotted over time, and means and standard error of the mean (SEM) were recorded.

### Statistical analysis

Data are presented in terms of relative frequencies. Inferential statistics including mean value (± SEM) and range were calculated for SNR, LLC, CER, and SI ratios. In this quantitative analysis, we tested the differences in SNR over time between all patients as well as the differences in CER, LLC, and SI ratios between HCCs, FNH lesions, and metastases on unenhanced, dynamic, and HP images with linear mixed models. The correlation structure over time was specified as autoregressive, and the pair-wise post-hoc comparisons were modified for multiplicity by means of Bonferroni adjustments. All reported p-values are two-sided, and a *P* value of 0.05 is considered to indicate a significant difference. All analyses were conducted with SAS 9.3 and the linear mixed model analyses with the procedure PROC MIXED. Descriptive statistics for LLC, CER, and SI ratios over time were calculated for hemangiomas and HCAs because of the small number of lesions.

## Results

**Figure 2:**
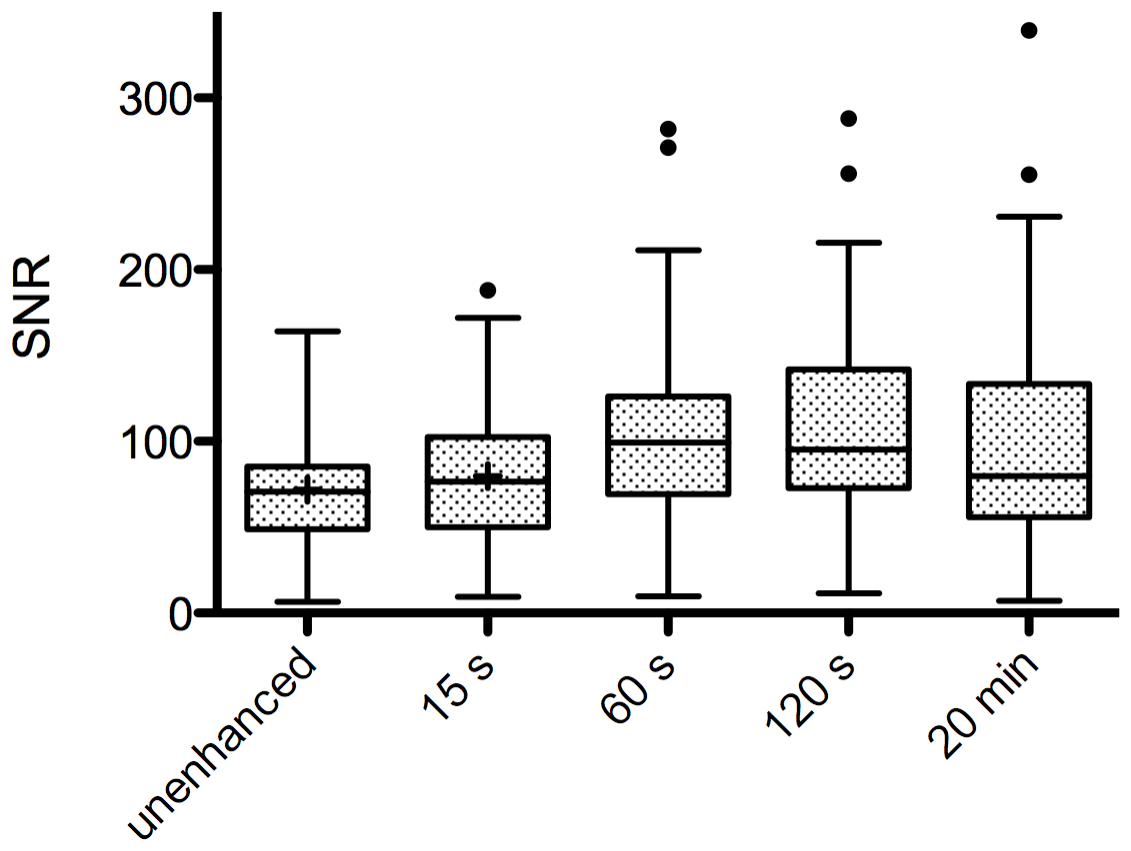
Signal-to-noise ratios (SNRs) of liver parenchyma in 83 patients. In arterial phase images 15-EOB-DTPA administration, a significant increase in SNR could be observed (mean ± SEM, 71.8±3.4 vs. 79.7±3.4; p = 0.028). Furthermore, a significant increase in SNR between 15 s and 60 s could be shown (mean ± SEM, 79.7±3.4 vs. 101.4±5.5; p<0.0001). Over the further time course, no other significant increase in SNR occurred (120 s, 108.0±5.7; 20 min, 98.6±6.6).

In contrast to the SNR of liver parenchyma of non-enhanced images (71.8±3.4), the SNR significantly increased after Gd-EOB-DTPA administration in all dynamic phases: in AP (79.7±4.0), in PVP (101.4±5.5), in LP (108.0±5.7), and in HP images after 20 min (98.6±6.6) (p<0.0001) (Fig 2).

The mean CERs of the HCCs were 45.3±5.0, 54.8±5.5, 49.7±4.7, and 31.1±8.5 at 15 s, 60 s, 120 s, and 20 min after contrast media injection. The CER did not significantly differ between the dynamic AP to LP (p = 0.087-0.361), but a significant difference in the CER was found between HP and PVP (p = 0.001) and HP and LP (p = 0.0011).

The corresponding CERs of FNH lesions (82.3±8.1, 88.1±8.6, 83.8±6.1, and 79.8±7.8) showed no significant difference between all dynamic phases over the time course (p = 0.472-0.614). Liver metastases (29.1±4.5, 52.8±4.8, 55.2±4.4, 15.1±6.8) differed significantly with regard to the CER between HP image and all dynamic phases over the time course (p≤0.049-0.001). The CER of adenomas (67.2±21.8, 71.9±2.4, 63.1±5.1, 21.4±13.0) showed a decrease in HP images, and the CER of hemangiomas (6.7±6.5, 29.6±15.5, 53.4±26.2, 53.7±26.4) continuously increased over the time course of dynamic imaging.

**Figure 3:**
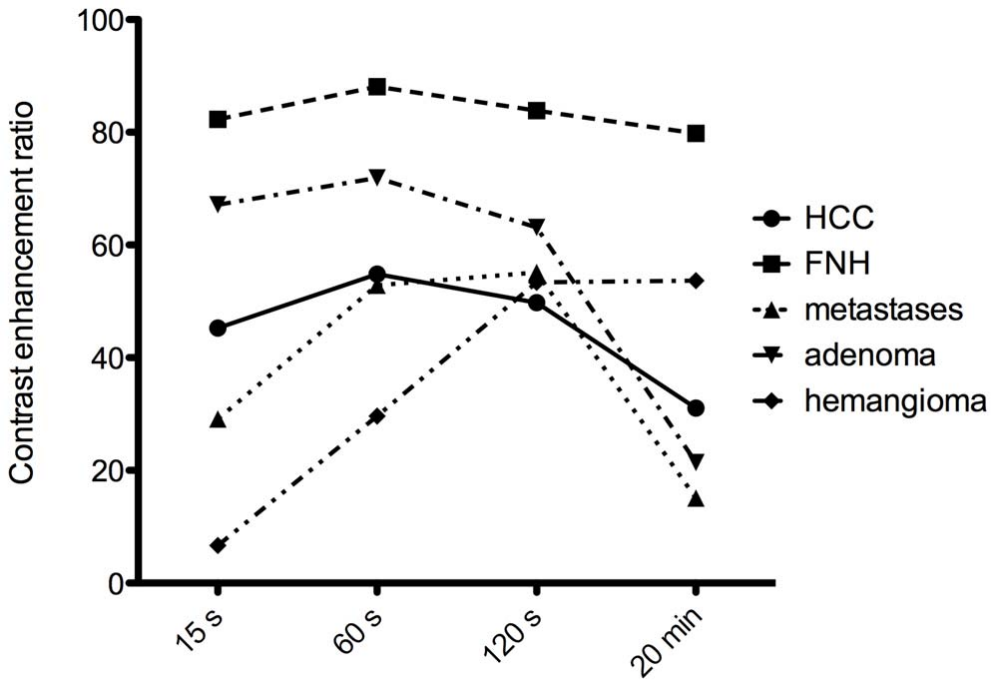
The graph shows the temporal mean contrast enhancement ratios (CERs) of HCCs, metastases, adenomas, hemangiomas, and FNH lesions at each MR imaging phase during the arterial phase (15 s), the portal venous phase (60 s), the late phase (120 s), and the hepatobiliary phase (20 min).

The temporal CER of HCCs, metastases, FNH lesions, adenoma and hemangioma at each MRI phase are shown in Fig. 3.

On T2-weighted images, the mean LLC values of all liver lesions were positive (HCCs 7.8±1.4, FNH lesions 4.6±1.5, metastases 8.7±2.8, HCAs 13.7±1.4, hemangiomas 23.2±4.3).

**Figure 4:**
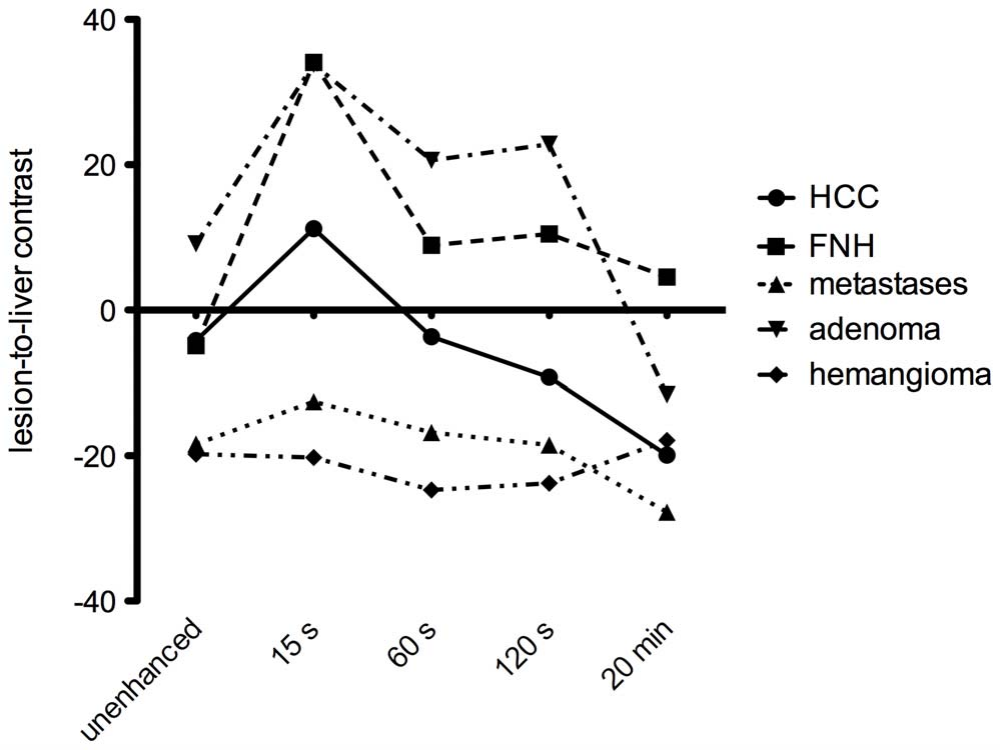
The graph shows the temporal mean liver-to-lesion contrast (LLC) ratios of HCCs, metastases, adenomas, hemangiomas, and FNH lesions at each MR imaging phase in unenhanced images, during the arterial phase (15 s), the portal venous phase (60 s), the late phase (120 s), and the hepatobiliary phase (20 min).

Fig. 4 shows the mean LLC values of all lesions on T1-weighted images over the time course after contrast media injection.

On unenhanced T1-weighted images, the LLC values of both HCCs and FNH lesions overlapped (p = 0.898). LCC values significantly differed between HCCs and metastases (p = 0.001) and between FNH lesions and metastases (p = 0.0151). With the exception of adenomas (9.1±7.9), all liver lesions showed negative LLC values on unenhanced T1-weighted images (HCCs, −4.2±3.2; FNH lesions, −4.9±2.1; metastases −18.4±2.3; hemangiomas, −19.8±2.7).

The mean LLC of FNH lesions during AP, PVP, LP, and HP images (AP, 34.1±3.8; PVP, 8.9±3.2; LP, 10.5±3.3; HP 4.4±4.2) were significantly higher than those of HCCs (AP; 11.2±3.6; PVP, −3.7±3.7; LP, −9.3±3.0; HP, −19.9±4.6; p≤0.0202–0.001) and metastases (AP, −12.5±2.8; PVP, −16.8±2.7; LP, −18.5±2.8; HP, −27.8±3.5; p<0.0001). The difference between the lesion and the surrounding liver parenchyma was significantly less for metastases than for HCCs (p≤0.0315–0.001) in all imaging phases except for the HP (p = 0.0684). The LLC of adenomas showed positive values in the AP (33.8±13.6), PVP (20.6±10.3), and LP (22.8±11.4) and decreased in the HP, showing a negative difference between the lesion and the surrounding liver parenchyma. LLC values of hemangiomas remained negative over the entire time course in the AP (−20,23±4.8), PVP (−24,71±3.2), LP (−23,80±7.3), and the HP (−17,89±7.6), showing hypointens lesions compared to the surrounding liver parenchyma.

In AP images, the SI ratio of FNH lesions (1.38±0.05) was significantly higher than that of HCCs (1.19±0.06) and metastases (0.87±0.02) (p = 0.0017; p<0.0001). Each lesion showed significantly decreased SI ratios (p<0.0001) in the HP images: FNH lesions, 1.05±0.03; HCCs, 0.8±0.04; metastases, 0.69±0.02.

The mean SI ratio of adenomas indicated hyperintensity in AP images and hypointensity in HP images, and the mean SI ratio of hemangiomas showed hypointensity in both the AP and the HP images.

**Figure 5:**
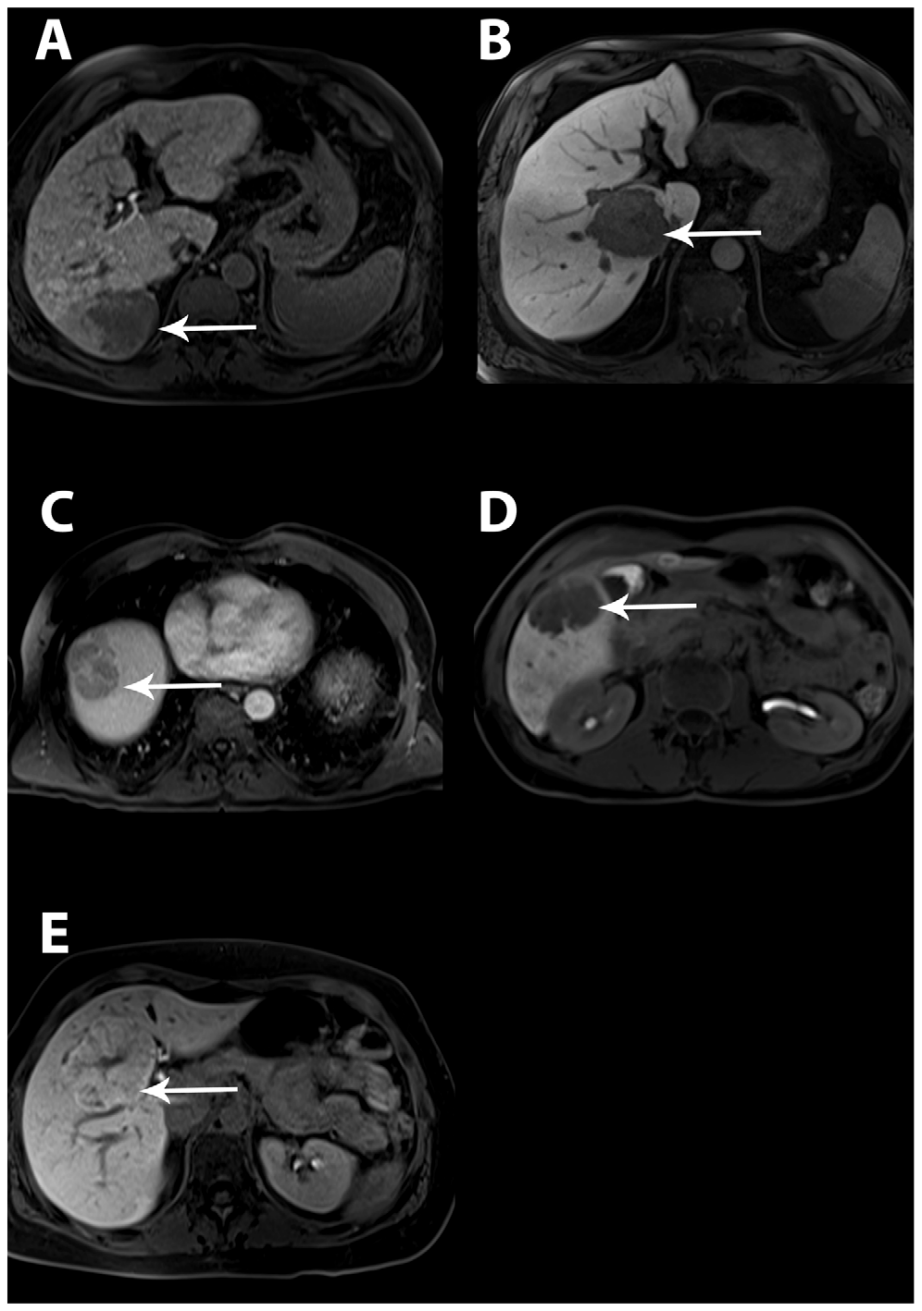
Transverse 3D fat-suppressed T1- weighted gradient-echo sequence obtained 20 min after Gd-EOB-DTPA administration in hepatobiliary phase: white arrows depict (A) hypointense HCC on cirrhotic liver parenchyma, (B) hypointense metastasis, (C) hypointense adenoma, (D) hypointense hemangioma, (E) hyperintense FNH lesion with central scar.

A representative hepatobiliary phase image of each benign and malignant liver lesion is shown in Fig. 5.

## Discussion

In clinical trials, liver-specific contrast media have been shown to markedly improve the detection of focal solid liver lesions [12], [13]. However, with regard to a possible preoperative evaluation of patients or the further management of patients after lesion detection, the consecutive diagnosis of focal solid liver lesions is of central importance in daily clinical routine. We therefore evaluated enhancement patterns of focal solid liver lesions in both dynamic phase and HP MR images; our images constitute a representative collection of liver lesions in the clinical routine of a university hospital.

In clinical trials, liver-specific contrast media have been shown to markedly improve the detection of focal solid liver lesions [12], [13]. However, with regard to a possible preoperative evaluation of patients or the further management of patients after lesion detection, the consecutive diagnosis of focal solid liver lesions is of central importance in daily clinical routine. We therefore evaluated enhancement patterns of focal solid liver lesions in both dynamic phase and HP MR images; our images constitute a representative collection of liver lesions in the clinical routine of a university hospital.

Enhancement patterns of liver lesions during dynamic phases after contrast media administration primarily depend on the vascularity and blood supply of a tumor. However, enhancement on delayed phase images is characterized by the cell specificity of MR contrast agents. Gd-EOB-DTPA is a liver-specific, hepatobiliary contrast agent that produces both dynamic perfusion and liver-specific hepatobiliary MR images. This way, Gd-EOB-DTPA combines the properties of an extracellular fluid contrast agent, such as Gd-DTPA, and a hepatobiliary agent, such as Mn-DPDP [14]. With regard to the detection and diagnosis of liver lesions, at least equal results could be obtained with Gd-EOB-DTPA and other extracellular contrast media in AP und PVP images [13]. During hepatocyte selective phases, Gd-EOB-DTPA is not only absorbed by normal liver parenchyma but also by focal solid liver lesions of hepatocellular origin (HCAs, FNH lesions, HCCs). The additional information provided by Gd-EOB-DTPA in HP images can help distinguish hepatocyte-containing lesions from non-hepatocyte-containing lesions [15], [16].

In our study population consisting of patients with metastases, benign liver lesions (FNH lesions, HCAs, hemangiomas), and HCCs, we observed considerable enhancement of liver parenchyma starting at 15 s after Gd-EOB-DTPA administration. Quantitative evaluation showed further significant enhancement of SNR during PVP and LP that has also been shown in other clinical trials [13] [17].

Comparing the SNR of patients with HCC (n = 34) and the SNR of patients with metastases and benign liver lesions (n = 49), a diminished SNR could be shown in the AP images (HCCs, 68.6±6.1 vs. non-HCCs, 87.5±5.2) and the HP images (HCCs, 89.5±9.3 vs. non-HCCs, 104.9±9.1) of patients with HCC. The transport of Gd-EOB-DTPA in hepatocytes is mediated by OATP1 and multidrug resistance-associated protein 2 (MRP2), and these two different transport systems are located at the sinusoidal and canalicular membranes of the cell [18]. Our findings support the results of other trials that showed delayed uptake of Gd-EOB-DTPA in patients with progressively impaired hepatic function, for example, in patients with liver fibrosis [19]. Decreased enhancement of cirrhotic liver parenchyma after Gd-EOB-DTPA administration has only recently been shown to be attributed to lower OATP1-activity, along with the slower contrast media hepatocyte uptake and the rapid elimination of Gd-EOB-DTPA due to up-regulated MRP2 activity [20].

Consistent to established imaging findings with Gd-EOB-DTPA and non-specific extracellular contrast media, HCCs in our trial became hyperintens with hyperenhancement in the AP after 15 s [13], [21], [22]. Over the further time course, we observed progressively negative differences between HCCs and the surrounding liver parenchyma during the PVP, the LP, and the HP with a most markedly negative contrast (washout) in the HP.

It has been shown that HCCs appear hypointens in HP images – most likely due to the down-regulation of OATP1 and the increased expression of MRP2 –, even though they lack the characteristic enhancement features of HCCs in early dynamic phases, for instance, due to the persistent portal venous blood supply in early HCC [23], [24]. Thus, Gd-EOB-DTPA might help differentiate small HCCs from dysplastic nodules and pseudovascular lesions, which do not commonly show hypointensity during the HP [25], [26]. In our trial, however, 8 HCCs appeared isointens to hyperintens in relation to the surrounding liver parenchyma, most likely because of the high MRP2 expression in the canalicular membrane and the down-regulation of MRP2 in the luminal membrane [27].

The CER of HCCs did not significantly change during the dynamic phases; however, the contrast enhancement significantly decreased during the HP, again emphasizing the added value of the HP in terms of characterizing HCCs.

Liver metastases appeared hyperintens on T2-weighted images and hypointens on unenhanced and dynamic phase images in relation to the surrounding liver parenchyma with a significant decrease of LLC between the dynamic LP and HP. FNH lesions and hepatic adenomas are focal solid liver lesions of hepatocellular origin, and a known characteristic feature of these lesions is the arterial enhancement during dynamic imaging [1], [28]. The correct differentiation of FNH lesions and hepatic adenomas is of great clinical relevance and has always been a challenge, because adenomas hold the possibility for malignant transformation and may cause severe complications, such as hemorrhage due to rupture of the adenoma [29].

FNH lesions, which typically appear as a solitary lesion in young women, are considered to consist of aggregated hepatocytes in terms of a proliferative response of liver parenchymal cells to a preexisting vascular malformation along with malformed biliary ducts [30]. It is therefore reasonable to assume that the presence of disordered vascular structures and malformed biliary ducts may lead to prominent arterial enhancement and to reduced biliary excretion of contrast media, resulting in signal hyperintensity during HP imaging. We observed strong arterial enhancement of all FNH lesions (100%) in AP images, and 7 FNH lesions (57.1%) were markedly hyperintens in relation to the surrounding liver parenchyma during the HP.

Hepatic adenoma, however, contain well-differentiated monotonous hepatocytes that are separated by dilated sinusoids lacking biliary elements or portal tracks and are normally surrounded by a stromal capsule [31]. In our trial, arterial enhancement was weaker in hepatic adenomas than in FNH lesions, most likely because of a dilutive effect of the contrast media in dilated sinusoids. Similar to FNH lesions, adenomas appeared hyperintens in AP, PVP, and LP images, impeding the differentiation between these two lesions. During the HP, however, adenomas appeared hypointens in comparison to the adjacent liver parenchyma. This finding supports other trials postulating that HP imaging may help differentiate FNH lesions and adenomas [10], [32], [33]. However, published data on enhancement patterns of hepatic adenomas with Gd-EOB-DTPA-enhanced HP MRI are very limited and do vary. To the best of our knowledge, only four trials exist describing the behavior of histologically proven adenomas in delayed Gd-EOB-DTPA-enhanced imaging: all adenomas described by Giovanoli et al. (3 adenomas) and Mohajer et al. (6 adenomas) showed hypointensity in HP imaging; out of the 3 adenomas investigated by Huppertz et al., 1 was hypointens, and 2 were hyperintens. In 2012, Grazioli et al. described 43 adenomas of which 40 showed hypointensity and only 3 hyperintensity during HP imaging, most likely owing to severe hepatic steatosis [9], [34]-[36]. Both the absence of biliary ductules and the altered expression of the uptake transporter organic anion transporting polypeptide (OATP 8) in adenomas may explain the lack of contrast uptake in HP images [6_ENREF_6,32].

Our trial has several limitations. First, we did not take a needle biopsy of every hepatic nodule. The majority of HCCs and metastases were proven by histology, diagnosed according to surgical findings or results of percutaneous biopsies. Because, in most cases, diagnosis of a benign liver lesion may lead to conservative management, FNH lesions, HCAs, and hemangiomas were not histologically confirmed. All benign lesions had remained unchanged at the follow-up examinations after more than 6 months without treatment.

Second, the low number of benign liver lesions in our trial necessitates further trials with a higher number of patients.

Furthermore, Gd-EOB-DTPA-enhanced MRI was conducted regardless of the liver function; delayed hepatocyte uptake concurs with diminished liver function, which might have influenced the SI of a lesion, particularly in case of HCC in cirrhosis.

Accepting these limitations we conclude that the clinical usefulness of Gd-EOB-DTPA is based on analyzing the biphasic enhancement characteristics of liver parenchyma and focal solid liver lesions during both the dynamic perfusion phases and the HP. Furthermore, Gd-EOB-DTPA-enhanced MRI may help diagnose focal solid liver lesions by evaluating their enhancement patterns.
